# Relationship between relative deprivation and health of Hainan Island residents: mediating effect of negative health behaviors

**DOI:** 10.7717/peerj.8728

**Published:** 2020-03-24

**Authors:** Na Wu, Anguo Fu, Zaisheng Zhang, Wuming He, Tianzeng Yao, Xuesong Sun, Zhiming Liao, Guanghui Hou

**Affiliations:** 1 School of Management, Hainan University, Haikou, China; 2 Department of Tourism Management, Hainan College of Economics and Business, Haikou, China; 3 College of Management and Economics, Tianjin University, Tianjin, China; 4 Lingnan Normal University, Department of Psychology & Guangdong Provincial Key Laboratory of Development and Education for Special Needs Children, Zhanjiang, China; 5 Shantou University Law School, Shantou, China

**Keywords:** Alcohol drinking, Smoking, Betel nuts chewing, Relative deprivation, Yitzhaki Index, Income distribution

## Abstract

Despite extensive evidence of the direct impact of relative deprivation on health, the mediating role of tobacco, alcohol and betel nuts in this impact has been largely ignored. This study aimed to verify whether these negative health behaviors are mediating factors for relative deprivation and health according to the mediating effect concept. Data from the Hainan Island Residents Health Interview Survey in 2017 were used. Variables including age, marital status, educational level, chronic diseases and area of residence were controlled for in multivariate analysis with separate sex analyses. Mediating effects of smoking, alcohol drinking and betel nut chewing, and whether the effects were complete or partial, were analyzed by logistic regression analysis. Smoking, alcohol drinking and betel nut chewing had a significant mediating effect in men, but not in women; however, alcohol drinking and betel nut chewing had similar, significant complete mediation in both sexes. Dissatisfaction following relative deprivation due to uneven income distribution may be relieved through these negative health behaviors. Therefore, better medical resources should be provided to improve residents’ health and the impact of income inequality on health, particularly the growing gap between the rich and poor, should be addressed.

## Introduction

Compared to non-smokers, smokers are more likely to suffer from various chronic diseases, such as asthma, lung cancer, laryngeal cancer and cardiovascular diseases (Doll et al., 2004; Ezzati & Lopez, 2003; Nogueira et al., 2018; Hayashida et al., 2010). Alcohol drinking is associated with more than 200 diseases (Clarke et al., 2017), especially increased incidence of liver cirrhosis, liver cancer and gastric ulcer (Bellis et al., 2016). Betel nut chewing not only directly leads to oral cancer (Lechner et al., 2019), but also induces other hazards, such as reproductive (Yuan et al., 2012) and hepatorenal (Jeng et al., 2014) toxicity and increased risk of cardiovascular diseases, chronic kidney diseases and type II diabetes mellitus, among others (Yuan et al., 2012; Jeng et al., 2014; Kampangsri et al., 2013). Previous studies have shown that the consumption of addictive commodities correlates with personal social and economic background factors. For example, men, people with low educational levels, low-income people, unemployed people and blue-collar workers tend to have addictive behaviors such as smoking, alcohol drinking and betel nut chewing (Becker, Grossman & Murphy, 1991; Benegal, Rajkumar & Muralidharan, 2008), which can produce a sense of happiness and cheerfulness but lead to addiction (Chu, 2001).

Although smoking, alcohol drinking, betel nut chewing and other unhealthy behaviors are harmful to health, once people encounter setbacks or are faced with pressure, they tend to seek relief and comfort in these unhealthy behaviors. According to the classic Relative Deprivation Theory, individuals evaluate their status and situation mainly through comparison with others. Members of vulnerable groups often experience the perception of being deprived of their basic rights, which not only causes them to lose many opportunities in real life, but also to experience more setbacks and pressures (Mummendey et al., 1999). Over time, as Wilkinson (1992) suggested, at a certain economic development level, income inequality results in comparison between people and those with relatively low income tend to develop negative psychological feelings of relative deprivation. Wilkinson (1996) further pointed out that dissatisfaction, depression, hostility and other negative emotions due to relative deprivation can easily lead to negative health behaviors, such as smoking, alcohol drinking and betel nut chewing, to obtain relief from pressure (Bellis et al., 2016; Benegal, Rajkumar & Muralidharan, 2008; Conway et al., 1981; Horwitz & Davies, 1994; Greitemeyer & Sagioglou, 2017; Pettigrew, 2016), and thus affect health.

The correlation between relative deprivation and personal health has received great attention from many scholars. The Yitzhaki Index, a relative deprivation index constructed by Yitzhaki (1979), is used in the analysis and measurement of relative deprivation. The Yitzhaki Index is calculated based on personal income. By implication, an individual can derive his/her individual relative deprivation by the sum of the income gaps between the individual’s own income and the incomes in a higher income comparison group, which is then divided by the total number of people in the comparison group. The higher the Yitzhaki Index, the stronger the relative deprivation due to the individual’s lower income. Ebert & Moyes (2000) have proven that the sum of the relative deprivation derived from each person’s Yitzhaki Index is equal to the product of the Gini coefficient by the social average income. This indicates that the Yitzhaki Index is directly proportional to the Gini coefficient (Yitzhaki, 1979). Furthermore, there is no better measurement method for relative deprivation known at present. Therefore, most articles in the literature have used this index to verify the hypothesis of relative deprivation.

Studies have reported that the higher a person’s Yitzhaki Index, the higher the risk of mental illness (Eibner, Sturn & Gresenz, 2004), poor health status, mortality rate, body mass index and health risk behaviors (Eibner & Evans, 2005). Studies by Kondo et al. (2008), Subramanyam et al. (2009) and Mangyo & Park (2011) also support the relative deprivation hypothesis, that is, the higher the relative deprivation, the worse the self-rated health status. Some studies have shown that individuals with a high relative deprivation may experience greater stress and depression, increasing the risks of heart disease, hypertension, eating disorders, drug abuse, suicide and death (Greitemeyer & Sagioglou, 2017; Pettigrew, 2016; Salti, 2010; Lhila & Simon, 2010). Thus, in the literature, relative deprivation due to unequal income distribution is receiving more attention. With the widening gap between the rich and poor, relative income is gradually replacing absolute income and has become an important factor affecting health.

Whether relative deprivation increases risk behaviors, such as smoking, alcohol drinking and betel nut chewing, has rarely been examined in the literature. Eibner & Evans (2005), as well as Kuo & Chiang (2013), found that individuals with higher relative deprivation are more likely to become current smokers. Siahpush et al. (2006) conducted an empirical analysis based on data from Australia and reported higher self-rated substance deprivation, higher subjective income inequality and lower social capital among smokers compared with non-smokers; however, smoking was not significantly correlated with relative deprivation (measured by the Yitzhaki Index) (Eibner, Sturn & Gresenz, 2004; Siahpush et al., 2006). Based on data from a Japanese population aged over 65 years, Kondo et al. (2009) found that unhealthy lifestyle habits, such as smoking and alcohol drinking, partially explained the correlation between higher relative deprivation and higher incidence of disability. Benegal, Rajkumar & Muralidharan (2008) reported that the proportions of people chewing betel nuts are higher in rural and low-income populations than in other groups, which may be due to the fact that the cost of betel nuts is lower than that of other psychoactive substances (such as cigarettes and alcohol).

Although the aforementioned literature took negative health behaviors into consideration, few reports existed regarding negative health behaviors as the transmission channel between relative deprivation and health status. Furthermore, Wilkinson (1996) pointed out that relative deprivation caused by lower income can easily lead to the release of anger and hostility through unhealthy risk behaviors such as smoking, alcohol drinking and betel nut chewing. Therefore, there may be direct and indirect impacts of relative deprivation, caused by income inequality, on health. Direct impact refers to stress resulting from relative deprivation that affects health directly, for example, by increasing the incidence of heart disease and hypertension (Kuo & Chiang, 2013). The indirect impact may result from dissatisfaction caused by comparison of individuals experiencing relative deprivation to others, which can then easily lead to relieving mental stress and enhancing self-satisfaction with negative health behaviors such as smoking, alcohol drinking and betel nut chewing, thus endangering health (Benegal, Rajkumar & Muralidharan, 2008; Chu, 2001; Wilkinson, 1992, 1996; Conway et al., 1981; Horwitz & Davies, 1994; Kuo & Chiang, 2013).

In summary, the direct impact of relative deprivation on health has received much attention from scholars and reports on issues related to relative deprivation have also focused on the impact of the Yitzhaki Index on physical and mental health and mortality or disability rate. Because direct effect models are simple and clear, the literature has thus far explored the impact of relative deprivation on health using the direct effect model. However, the direct effect model ignores the mediating role of tobacco, alcohol and betel nuts in the impact of relative deprivation on health. We propose a new perspective that decreasing income inequality may be a better way to reduce the consumption of addictive substances such as tobacco, alcohol and betel nuts. Based on Hainan Province-wide survey data and the mediating effect concept put forward by Baron & Kenny (1986), this study verified whether the three negative health behaviors of smoking, alcohol drinking and betel nut chewing played a mediating role in the influence of relative deprivation on health status. Because men and women may have different degrees of these unhealthy behaviors, this study estimated effect in men and women separately and evaluated whether the mediating effect differed by sex.

## Materials and Methods

### Selection of data sources and study samples

This study used data from the island-wide Health Interview Survey of Hainan Island Residents (HISHIR), conducted by the Health Commission of Hainan Province in 2017. To ensure island-wide representativeness of the study subjects, a multi-stage stratified sampling system was adopted. The 2017 HISHIR was based on the Hainan Province population from household registration by the end of 2016. The extraction rate from each stratum in counties and cities satisfied the probability proportional to size sampling method and 22,225 respondents were extracted phase-by-phase from cities, counties, villages and towns and communities and individuals. Finally, 19,334 respondents completed the interview survey, with a completion rate of 87.0%. Three types of questionnaires were used for age categories as follows: questionnaires for those aged <12, ≥12 and <65 and ≥65 years. The Health Commission of Hainan Province used “sex” and “age” as two variables to perform consistency tests between the interviewed subjects and general population data. All test results showed no significant difference, indicating that the subjects in this survey were representative (Health Commission of Hainan Province (China), 2017). Because those aged 12–24 years were in school and the effects of negative behaviors on health manifest after a period of time, this study included 11,491 subjects aged ≥25 years and <65 years. Those excluded were 432 students and 632 people who were unable to work due to health or health-related problems, because the information on relative deprivation may be missing due to the lack of personal income. Missing values and variables with incomplete data (93) were also deleted, resulting in the inclusion of data from a total of 10,334 subjects, among whom 5,601 were men and 4,733 were women.

The questionnaires included basic personal information (age, sex, educational level, marital status, home environment and area of residence), self-rated health status (chronic disease history, family history, various disease states and physical functions) and utilization of medical services and preventive health care (utilization and expenditure on Western medicine, traditional Chinese medicine, dental clinics, emergency care and hospitalization, as well as awareness and utilization of preventive health care). Other information included personal health behaviors (smoking, alcohol drinking, betel nut chewing, physical activity, nutrition and dietary style), home financial status and insurance status, etc.

### Testing for the mediating effect

According to Baron and Kenny’s method of testing for the mediating effect (Baron & Kenny, 1986), the impact of relative deprivation on health was first predicted; if a significant correlation was found between the two, then the effect of relative deprivation on smoking, alcohol drinking and betel nut chewing was examined. If there was a significant correlation again between these, finally, the effect of relative deprivation, smoking, alcohol drinking and betel nut chewing on health was predicted simultaneously. If relative deprivation showed a significant effect from smoking, alcohol drinking and betel nut chewing, but relative deprivation showed no significant effect on health (i.e., the direct impact path of the independent variables on the outcome variables was not significant after the mediating variables were included), then smoking, alcohol drinking and betel nut chewing (the mediating variables) had complete mediation. If relative deprivation had a significant effect on smoking, alcohol drinking and betel nut chewing, but the relative deprivation had a significant effect on health with reduced significance (i.e., the direct impact path of the independent variables on the outcome variables was significant, but the coefficient decreased after inclusion of the mediating variables), then smoking, alcohol drinking and betel nut chewing had partial mediation.

### Empirical model and variable setting

(1) $${\rm Step}\; 1\colon {\rm Health}={\rm{\alpha}}_{0}+{\rm{\alpha}}_{1}{\rm RD}+{\rm{\alpha}}_{2} X+{\rm{\varepsilon}}_{1}$$

(2) $${\rm Step}\; 2\colon {\rm Bad} \; {\rm behavior} \; = {\rm \beta} _{0}+ {\rm\beta} _{1} {\rm RD}+ {\rm \beta}_{2} X + {\rm \varepsilon}_{2}$$

(3) $${\rm Step}\;3\colon{\rm Health}={\rm{\gamma}} _{0}+{\rm{\gamma}} _{1}{\rm RD}+{\rm{\gamma}} _{2}{\rm Bad\ behavior}+{\rm{\gamma}} _{3} X + {\rm{\varepsilon}} _{3}$$

where, Health, Self-rated health status; Bad behavior, Personal negative health behaviors of smoking, alcohol drinking, and betel nut chewing; RD, Degree of individual’s relative deprivation; *X*, Personal socio economic and demographic variables; ε_1_, ε_2_, ε_3_, error term.

In this study, “personal self-rated health” was regarded as a health measurement indicator. The assessment was made based on the questionnaire item: “Generally speaking, what do you think of your current health?” Five, four, three, two and one point was assigned to “excellent”, “very good”, “good”, “normal” and “not good”, respectively; the higher the score, the better the self-perceived current health status. A previous study found that “personal self-rated health status” has high predictive power for mortality and is highly correlated with many objective health indicators (Jones & Wildman, 2008). With reference to the classification method proposed by Mohr et al. (2013), alcohol drinking behavior was classified based on the frequency of drinking and alcohol addiction, which was evaluated using the 10-item Brief Michigan Alcoholism Screening Test (BMAST). Accordingly, alcohol drinking behavior was categorized into “never drink”, “have not drunk in the past year”, “have drunk in the past year but do not have an addiction” and “have drunk in the past year and possibly have an addiction” (Mohr et al., 2013).

Because there is still no specific basis for the evaluation of smoking behavior and style or smoking addiction, the four categories of Mohr et al.’s (2013) classification of smoking behavior were used: “never smoke”, “have not smoked in the past year”, “have smoked in the past year but not every day” and “have smoked every day in the past year”, according to the smoking frequency. Regarding betel nut chewing behavior, Fan, Nie & Chen (2019) suggested four categories: “never chew”, “have not chewed in the past year”, “have chewed in the past year but not every day” and “have chewed every day in the past year”.

To derive the personal negative health behavior variable (Bad behavior), the respondents’ answers to the questions about smoking, alcohol drinking and betel nut chewing were used as the basis for assessment and classified into different addiction degrees as follows: according to the questions “From the past till now, have you smoked more than five packs of cigarettes?” and “Do you smoke every day, sometimes, or never?”; the degree of individual addiction to smoking was assigned 0, 1, 2 or 3 points for “never smoked”; “once smoked but do not currently smoke”; “currently smoke every day”; or “currently smoke but not every day,” respectively. According to the questions, “Have you ever drunk alcohol?”, “How often have you drunk in the past year?”, the 10-item (BMAST), and other questionnaire items, the individuals’ degree of addiction to alcohol drinking was divided into four grades: 0, 1, 2 or 3 points for “never drink”; “once drank but have not drunk in the past year”; “have drunk in the past year but do not have an addiction”; and “have drunk in the past year and possibly have an addiction”, respectively. Betel nut chewing was classified using the questionnaire items “Do you chew betel nuts?” and “How many have you chewed in the past week?” into four grades, 0, 1, 2 or 3 points for “never chew”; “once chewed but not chewing currently”; “chewing currently but not every day”; and “chewing every day currently”, respectively.

Only 1.58% of women were “currently smoking every day”, and only 0.84% and 0.67% of women “have drunk in the past year and possibly have an addiction” or were “chewing currently but not every day”, respectively. In order to avoid small numbers in some categories, women who “never smoked” or “once smoked but not smoking currently” were merged into “current non-smokers”; those who were “currently smoking every day” and “currently smoking but not every day” were merged into “current smokers”; those who “never drink” and “once drank but had not drunk in the past year” were merged into “current non-drinkers”; whereas those who “have drunk in the past year but do not have an addiction” and “have drunk in the past year and possibly have an addiction” were merged into “current drinkers.” Furthermore, those who “never chewed” and “once chewed but not chewing currently” were merged into “current non-chewers”, whereas those who were “chewing currently but not every day” and “chewing every day currently” were merged into “current chewers”.

From individuals’ answers to the questionnaire item, “How much was your average monthly income (including various types of income, such as investment income, children’s bestowal, social assistance and pension) in the past year?” the Yitzhaki Index was calculated. Average income options included “no income”; “less than RMB 1,500 yuan” (1 yuan is equivalent to 0.1399 USD); “RMB 1,500–2,999 yuan”; “RMB 3,000–4,499 yuan”; “RMB 4,500–5,999 yuan”; “RMB 6,000–7,499 yuan”; “RMB 7,500–8,999 yuan”; “RMB 9,000–14,999 yuan”; “RMB 15,000–20,999 yuan”; and “RMB 21,000 yuan and above”. Due to the ordinal nature of this variable, the median value of each income interval was used to represent the actual income for that interval (Kuo & Chiang, 2013). For respondents with “RMB 21,000 yuan and above”, reference was made to the 2015 Chinese General Social Survey for an estimation (Chinese National Survey Data Archive, 2015), and RMB 33,000 yuan represented the personal income. We divided the calculated Yitzhaki Index by 3,000 to make the estimation coefficient easy to interpret (Eibner & Evans, 2005; Kondo et al., 2008, 2009; Subramanyam et al., 2009; Kuo & Chiang, 2013).

Personal socioeconomic and demographic variables (*X*), such as age, marital status, educational level, chronic diseases, hometown and area of residence, were controlled for in the analysis. The respondents were divided into four age groups: 25–34, 35–44, 45–54 and 55–64 years. The reference group was the 55–64 years’ age group. Marital status was divided into “with partner” (including married and living with spouse and unmarried cohabitation) and “without partner” (including unmarried, divorced, living separately and widowed; reference group). Educational level was divided into four groups: “primary school (inclusive) or below” (reference group), “junior high school”, “high school or technical secondary school”, and “junior college, undergraduate, or above”. Chronic diseases were divided into “with chronic diseases” (experiencing hypertension, diabetes, hyperlipidemia, heart disease, or other chronic diseases) and “without chronic diseases” (not experiencing any of these; reference group). Area of residence was divided into “rural area” (reference group), “township”, “county” and “city”. Each county has jurisdiction over several townships, each township has jurisdiction over several villages and all villages are collectively referred to as rural areas.

Apart from defining the reference groups for all variables, this study assumed that individuals compared themselves to those of the same sex, age and educational levels. Therefore, combinations of these three variables were used to define the comparison groups in eight categories (all subjects, sex, age, educational level, sex + age, sex + educational level, age + educational level and sex + age + educational level). In this study, the male and female subjects were evaluated separately. Therefore, in the comparison groups, all subjects, age, educational level and age + educational level were used for the estimation. Wilkinson suggested that individuals’ comparison objects may cross areas of residence due to mass media (Wilkinson, 1996). However, people are likely to compare their differences in resources with others in the same situation and culture, for example, differences exist between people despite the same income, because people living in villages or cities differ greatly in their relative deprivation. Thus, this study also included the area of residence in the comparison groups, totaling five comparison groups, in addition to the above four.

Although the values assigned to the variables’ responses: (1) (2) and (3) are ordinal with a preferred order, Greene (2003) showed in the Ordered Probit Model that such options (such as 1, 2, 3, 4 or 5) are only the preferred order; difference among the options is not necessarily the same and the value of the options does not represent the actual value. Hence, the use of the Ordered Probit Model for calculating the point estimate, when a categorical variable with a sequence is to be interpreted. See Table 1 for the descriptive statistics of the subjects’ characteristics.

**Table 1:** Descriptive statistics of the subjects’ characteristics (*n* = 10,334). “Self-perceived health status” and “personal negative health behavior” among the explained variables and “degree of individual relative deprivation” among the explanatory variables are reported as “mean values”; the remaining explanatory variables are all reported in percentages. However, the classifications under “self-perceived health status” (not good, normal, good, very good and excellent), “smoking” and “alcohol drinking” are percentages.

Variable	Males (*n* = 5,601)		Females (*n* = 4,733)	
	%	Mean	%	Mean
**Explained variables**				
*Self-perceived health status*		2.78		2.72
Not good	4.7		4.1	
Normal	34.7		36.2	
Good	31.6		33.5	
Very good	25.6		23	
Excellent	3.4		3.2	
**Personal negative health behavior**				
*Smoking*		1.63		0.21
Never smoke	35.5		94.1	
Once smoked but not smoking now	16.6		(Current non-smokers)	
Smoking now but not every day	5.3		5.9	
Smoking every day now	42.6		(Current smokers)	
*Alcohol drinking*		1.54		0.83
Never drink	23.2		65.8	
Once drank but have not drunk in the past year	11.4		(Current non-drinkers)	
Have drunk in the past year but no addiction	60.3		34.2	
Have drunk in the past year and possibly have addiction	5.1		(Current drinkers)	
*Betel nuts chewing*		1.73		0.47
Never chew	16.1		67.2	
Once chewed but not chewing now	18.5		(Current non-chewers)	
Chewing now but not every day	39.8		32.8	
Chewing every day now	25.6		(Current chewers)	
**Explanatory variable**				
*Degree of individual relative deprivation*				
All subjects		1.38		1.01
Age (years)		1.23		0.86
Educational level		1.35		0.97
Area of residence		1.40		1.04
Age + educational level		1.16		0.91
*Age (years)*				
25–34	29.1		31.2	
35–44	25.6		28.2	
45–54	27.5		27.4	
55–64	17.8		13.2	
*Marital status*				
With partner	64.7		65.3	
Without partner	35.3		34.7	
*Educational level*				
Primary school	18.3		19.7	
Junior high school	28.4		32.4	
Normal high school or technical secondary school	35.2		36.2	
Junior college or undergraduate	18.1		11.7	
*Chronic diseases*				
With chronic diseases	35.3		29.8	
Without chronic diseases	64.7		70.2	
*Area of residence*				
Rural area	36.3		37.3	
Township	31.1		30.4	
County	21.4		22.1	
Urban area	11.2		10.2	

### Ethical approval and consent to participate

This study was conducted in accordance with the Declaration of Helsinki and the protocol was reviewed and approved by the ethics committee of Hainan University before the study (Ethical Approval Code: 20171203006). At the beginning of the survey, all the people involved in the study were informed of the purpose and content of the survey, and they signed the consent form or their fingerprints were taken.

## Results

### Descriptive statistics of the subjects’ characteristics

Table 1 shows that the average self-perceived health status of men was 2.78, slightly higher than the 2.72 in women. The degrees of men’s addiction, compared to women’s, were significantly higher, especially for smoking (*M*_male_ = 1.63 vs. *M*_female_ = 0.21) and betel nut chewing (*M*_male_ = 1.73, *M*_female_ = 0.47). Five degrees of relative deprivation, calculated by Yitzhaki Index, were reported. The higher the category in the comparison group, the lower the average Yitzhaki Index. The higher number of categories set for the comparison groups led to smaller differences among individuals; therefore, the lower the relative deprivation of individuals, the lower the calculated Yitzhaki Index. Men had higher relative deprivation than women, which means that the income distribution difference among men was greater than that among women.

Among the 10,334 study subjects included in this analysis, men and women comprised 54.2% and 45.8%, respectively. The age structures of men and women were similar, with the lowest and relatively higher proportions in the 55–64 and 25–34 years age group, respectively. Respondents with partners comprised approximately 65% and there was little difference in marital status between men and women. The educational level of men was slightly higher than that of women. Approximately 18% of men had a bachelor’s or junior college degree, whereas approximately 12% of women did. Experience of any chronic disease (hypertension, diabetes, hyperlipidemia or heart disease) was reported in 35% of men, higher than the 30% in women. More than 35% of the study subjects lived in rural areas, followed by townships and counties, whereas those living in urban areas were the least, at about 10%.

### Regression analyses

As shown in Table 2, regardless of the comparison group, whether all subjects, age, educational level, area of residence, or age + educational level, a significantly negative correlation occurred between relative deprivation and health status in both men and women. From the first step of the analysis, results with significant coefficients were obtained. Therefore, the second step of the analysis on the effect of relative deprivation on smoking, alcohol drinking and betel nut chewing was performed. For smoking behavior, as shown in Table 3, the coefficient in men was positively correlated regardless of the comparison group. However, for women, there was a significant correlation only when the comparison group was set as educational level and the level of significance was lower than that in men. For alcohol drinking behavior, as shown in Table 4, under any comparison group, the estimated coefficients in both men and women were positively correlated. For betel nut chewing behavior, as shown in Table 5, the estimation coefficients for men and women were positively correlated under any comparison group.

**Table 2:** First regression results of health status.

Name of variable	Males (*n* = 5,601)					Females (*n* = 4,733)				
Explanatory variable										
Degree of individual relative deprivation										
All subjects	−0.13***					−0.09***				
Age (years)		−0.13***					−0.07***			
Educational level			−0.12***					−0.07***		
Area of residence				−0.13***					−0.09	
Age + educational level					−0.10***					−0.07***
Control variable										
Age (years)										
25–34	0.28***	0.23***	0.29***	0.28***	0.23***	0.14**	0.20***	0.13**	0.14**	0.15**
35–44	0.16***	0.24***	0.16***	0.16***	0.16***	0.12**	0.21***	0.15**	0.15**	0.15**
45–54	0.10*	0.17***	0.09*	0.10*	0.09*	0.13**	0.18***	0.13***	0.14***	0.13***
55–64 (Ref. group)										
Marital status										
With partner	0.12***	0.11**	0.10***	0.12***	0.13***	0.04	0.05	0.04	0.05	0.05
Educational level										
Primary school and lower (Ref. group)										
Junior high school	0.04	0.03	0.18**	0.03	0.12^+^	0.03	0.06	0.04	0.06	0.07
Normal high school or technical secondary school	0.16**	0.16**	0.25***	0.16**	0.29***	0.20***	0.21***	0.27***	0.21***	0.25***
Junior college or undergraduate	0.23***	0.23***	0.44***	0.27***	0.49***	0.23***	0.28***	0.37***	0.25***	0.38***
Chronic diseases										
With chronic diseases	−0.48***	−0.47***	−0.48***	−0.48***	−0.46***	−0.43***	−0.42***	−0.43***	−0.43***	−0.41***
Area of residence										
Rural area (Ref. group)										
Township	−0.01	−0.02	−0.01	−0.01	−0.01	−0.02	−0.01	−0.01	−0.02	−0.01
County	−0.05^+^	−0.06^+^	−0.05^+^	−0.06^+^	−0.05^+^	−0.15***	−0.15***	−0.14***	−0.13***	−0.13***
Urban area	0.07	0.06	0.04	0.06	0.05	0.02	0.03	0.04	0.02	0.01

**Notes:**

* *p* < 0.05.

** *p* < 0.01.

*** *p* < 0.001.

+ *p* < 0.1.

Ref, reference.

**Table 3:** Regression results of smoking.

Name of variable	Males (*n* = 5,601)						Females (*n* = 4,733)					
Explanatory variable												
Degree of individual relative deprivation												
All subjects	0.08***						0.05					
Age (years)		0.07***						0.07				
Educational level				0.06***						0.07*		
Area of residence					0.06***						0.05	
Age + educational level						0.08***						0.05
Control variable												
Age (years)												
25–34	0.48***	0.48***		0.48***	0.46***	0.57***	0.96***	0.96***		0.97***	0.98***	1.05***
35–44	0.59***	0.57***		0.65***	0.64***	0.64***	0.64***	0.59***		0.66***	0.66***	0.64***
45–54	0.40***	0.37***		0.37***	0.38***	0.38***	0.26*	0.18		0.26*	0.23*	0.21^+^
55–64 (Ref. group)												
Marital status												
With partner	−0.16***	−0.14***		−0.16***	−0.14***	−0.15***	−0.68***	−0.67***		−0.67***	−0.67***	−0.68***
Educational level												
Primary school and lower (Ref. group)												
Junior high school	0.17*	0.17*		0.07	0.17*	0.10	0.07	0.07		0.07	0.08	0.06
Normal high school or technical secondary school	−0.14*	−0.14*		−0.18**	−0.13*	−0.21***	−0.09	−0.10		−0.15	−0.07	−0.19^+^
Junior college or undergraduate	−0.82***	−0.82***		−0.88***	−0.82***	−0.93***	−1.06***	−1.06***		−1.19***	−1.06***	−1.21***
Chronic diseases												
With chronic diseases	−0.19***	−0.18***		−0.17***	−0.18***	−0.19***	−0.05	−0.04		−0.03	−0.05	−0.04
Area of residence												
Rural area (Ref. group)												
Township	−0.02	−0.01		−0.01	−0.02	−0.01	−0.36***	−0.36***		−0.35***	−0.37***	−0.38***
County	−0.01	−0.02		−0.01	−0.02	−0.01	−0.41***	−0.41***		−0.37***	−0.41***	−0.41***
Urban area	0.07	0.09		0.08	0.08	0.09	−0.01	−0.03		−0.03	−0.03	−0.02

**Notes:**

* *p* < 0.05.

** *p* < 0.01.

*** *p* < 0.001.

+ *p* < 0.1.

Because of the low correlation (<0.3) among smoking, alcohol drinking, and betel nut chewing in men and women, these variables were not controlled for in the multivariate analysis.

Ref, reference.

**Table 4:** Regression results of alcohol drinking.

Name of variable	Males (*n* = 5,601)					Females (*n* = 4,733)				
Explanatory variable										
Degree of individual relative deprivation										
All subjects	0.12***					0.08***				
Age (years)		0.11***					0.09***			
Educational level			0.13***					0.11***		
Area of residence				0.10***					0.09***	
Age + educational level					0.10***					0.08***
Control variable										
Age (years)										
25–34	0.27***	0.26***	0.27***	0.28***	0.19**	0.25***	0.31***	0.27***	0.25***	0.25**
35–44	0.25***	0.30***	0.25***	0.24***	0.24***	0.24**	0.27***	0.24***	0.23***	0.24**
45–54	0.07	0.12*	0.08^+^	0.10^+^	0.09^+^	0.20***	0.29***	0.22***	0.23***	0.24***
55–64 (Ref. group)										
Marital status										
With partner	0.05	0.08	0.06	0.06	0.05	−0.28***	-0.27***	−0.25***	−0.28***	−0.25***
Educational level										
Primary school and lower (Ref. group)										
Junior high school	0.22**	0.21***	0.38***	0.22***	0.28***	0.24***	0.25***	0.23***	0.25***	0.26***
Normal high school or technical secondary school	0.17*	0.15*	0.26***	0.17*	0.28***	0.26***	0.24***	0.32***	0.25***	0.33***
Junior college or undergraduate	0.07	0.06	0.25***	0.08	0.28***	0.31***	0.30***	0.38***	0.39***	0.42***
Chronic diseases										
With chronic diseases	0.03	0.02	0.02	0.02	0.01	0.04	0.05	0.04	0.05	0.05
Area of residence										
Rural area (Ref. group)										
Township	−0.03	−0.02	−0.04	−0.02	−0.02	−0.14**	−0.12**	−0.15**	−0.13**	−0.15**
County	−0.16***	0.17 ***	−0.17***	−0.16***	−0.17***	−0.33***	−0.35***	−0.35***	−0.33***	−0.35***
Urban area	0.18**	0.16**	0.15**	0.17**	0.16**	0.01	0.02	0.01	0.02	0.02

**Notes:**

* *p* < 0.05.

** *p* < 0.01.

*** *p* < 0.001.

+ *p* < 0.1.

Because of the low correlation (<0.3) among smoking, alcohol drinking, and betel nut chewing in men and women, these variables were not controlled for in the multivariate analysis.

Ref, reference.

**Table 5:** Regression results of betel nuts chewing.

Name of variable	Males (*n* = 5,601)					Females (*n* = 4,733)				
Explanatory variable										
Degree of individual relative deprivation										
All subjects	0.07***					0.06***				
Age (years)		0.06***					0.06***			
Educational level			0.05***					0.07***		
Area of residence				0.06***					0.07***	
Age + educational level					0.10***					0.08***
Control variable										
Age (years)										
25–34	0.49***	0.47***	0.45***	0.47***	0.53***	0.97***	0.96***	0.97***	0.99***	1.01***
35–44	0.64***	0.59***	0.63***	0.64***	0.66***	0.65***	0.57***	0.65***	0.64***	0.64***
45–54	0.37***	0.36***	0.38***	0.40***	0.37***	0.25*	0.18	0.25*	0.25*	0.22^+^
55–64 (Ref. group)										
Marital status										
With partner	−0.16***	−0.15***	−0.15***	−0.16***	−0.14***	−0.66***	−0.68***	−0.66***	−0.67***	−0.68***
Educational level										
Primary school and lower (Ref. group)										
Junior high school	0.17*	0.17*	0.07	0.17*	0.12	0.07	0.07	0.06	0.08	0.04
Normal high school or technical secondary school	−0.12*	−0.12*	−0.18**	−0.15*	−0.23***	−0.09	−0.08	−0.17	−0.08	−0.19^+^
Junior college or undergraduate	−0.78***	−0.78***	−0.93***	−0.80***	−0.93***	−1.06***	−1.06***	−1.25***	−1.07***	−1.21***
Chronic diseases										
With chronic diseases	−0.17***	−0.17***	−0.20***	−0.20***	−0.19***	−0.05	−0.04	−0.05	−0.03	−0.05
Area of residence										
Rural area (Ref. group)										
Township	−0.02	−0.01	−0.02	−0.02	−0.01	−0.38***	−0.35***	−0.37***	−0.37***	−0.35***
County	−0.01	−0.01	−0.01	−0.02	−0.03	−0.38***	−0.38***	−0.36***	−0.42***	−0.42***
Urban area	0.09	0.07	0.08	0.08	0.07	−0.03	−0.02	−0.04	−0.01	−0.03

**Notes:**

* *p* < 0.05.

** *p* < 0.01.

*** *p* < 0.001.

+ *p* < 0.1.

Because of the low correlation (<0.3) among smoking, alcohol drinking, and betel nut chewing in men and women, these variables were not controlled for in the multivariate analysis.

Ref, reference.

After the second step of the analysis, the regression analysis results showed that the coefficients of smoking, alcohol drinking and betel nut chewing in men and alcohol drinking and betel nut chewing in women were significant. Therefore, the third step was carried out to predict the impact of relative deprivation, smoking, alcohol drinking and betel nut chewing on health and to analyze whether smoking, alcohol drinking and betel nut chewing have complete or partial mediation on the impact of relative deprivation on health. Table 6 shows that, when the comparison group was set as all subjects for men and women, respectively, there was a negative correlation between relative deprivation and health status. Compared with the results shown in Table 2, the coefficients and level of significance both decreased. In terms of the mediating variables, for men, only when the comparison groups were set as all subjects and age, the three unhealthy mediating variables (smoking, alcohol drinking and betel nut chewing) showed partial mediation. When educational level, area of residence and age + educational level were set as the comparison groups, the three unhealthy variables showed complete mediation. For women, smoking showed complete mediation only when the comparison group was set as educational level. When the comparison group was set as all subjects, partial mediation was shown. When the comparison groups were set as age, educational level, area of residence and age + educational level, complete mediation was shown.

**Table 6:** Second regression results of health status.

Name of variable	Males (*n* = 5,601)					Females (*n* = 4,733)				
Explanatory variable										
Degree of individual relative deprivation										
All subjects	−0.12*					−0.10***				
Age (years)		−0.11*					−0.05			
Educational level			−0.09					−0.04		
Area of residence				−0.09					−0.06	
Age + educational level					−0.02					−0.02
Personal negative health behavior										
Smoking	−0.03*	−0.02*	−0.02*	−0.03*	−0.03*	−0.13*	−0.14*	−0.14*	−0.15*	−0.14*
Alcohol drinking	−0.09***	−0.10***	−0.09***	−0.09***	−0.10***	−0.02*	−0.02*	−0.03*	−0.04*	−0.02*
Betel nuts chewing	−0.13***	−0.12***	−0.12***	−0.13***	−0.12***	−0.10***	−0.10***	−0.09***	−0.08***	−0.09***
Control variable										
Age (years)										
25–34	0.25***	0.23***	0.26***	0.27***	0.27***	0.14**	0.18***	0.17**	0.14**	0.12**
35–44	0.16***	0.27***	0.17***	0.18***	0.18***	0.15**	0.19***	0.14**	0.15**	0.14**
45–54	0.08*	0.18***	0.10*	0.10*	0.09*	0.15**	0.20***	0.14***	0.14***	0.15***
55–64 (Ref. group)										
Marital status										
With partner	0.10***	0.11**	0.12***	0.10***	0.11***	0.04	0.05	0.04	0.05	0.05
Educational level										
Primary school and lower (Ref. group)										
Junior high school	0.04	0.03	0.18**	0.03	0.12^+^	0.05	0.06	0.05	0.05	0.06
Normal high school or technical secondary school	0.16**	0.16**	0.25***	0.15**	0.28***	0.18***	0.21***	0.24***	0.21***	0.25***
Junior college or undergraduate	0.25***	0.25***	0.46***	0.27***	0.48***	0.26***	0.27***	0.37***	0.25***	0.36***
Chronic diseases										
With chronic diseases	−0.48***	−0.47***	−0.48***	−0.48***	−0.48***	−0.43***	−0.42***	−0.42***	−0.43***	−0.43***
Area of residence										
Rural area (Ref. group)										
Township	−0.01	−0.02	−0.01	−0.01	−0.02	−0.02	−0.01	−0.01	−0.01	−0.01
County	−0.05^+^	−0.06^+^	−0.06^+^	−0.04^+^	−0.06^+^	−0.15***	−0.15***	−0.14***	−0.13***	−0.13***
Urban area	0.05	0.05	0.06	0.05	0.04	0.02	0.02	0.02	0.03	0.03

**Notes:**

* *p* < 0.05

** *p* < 0.01.

*** *p* < 0.001.

+ *p* < 0.1.

Ref, reference.

## Discussion

According to China’s National Bureau of Statistics survey data, the Gini coefficient in mainland China doubled and exceeded the warning level, from 0.23 in 1980 to about 0.47 in 2017 (National Bureau of Statistics, 2018). This shows an increasingly uneven income distribution. Scholars have long accorded importance to the impact of income inequality on health. Most studies support the negative impact of income inequality on health (Eibner & Evans, 2005; Kondo et al., 2008; Subramanyam et al., 2009; Babones, 2008). The impact of relative deprivation, caused by income inequality, on health has recently attracted research attention, with results showing that the higher the relative deprivation due to income inequality, the greater the negative impact on health (Eibner, Sturn & Gresenz, 2004; Eibner & Evans, 2005; Kondo et al., 2008, 2009; Subramanyam et al., 2009; Mangyo & Park, 2011). In contrast to previous studies, this study used the concept of mediating effects proposed by Baron & Kenny (1986) and a large sample size to verify whether smoking, alcohol drinking and betel nut chewing are mediating factors for the hypothesis of relative deprivation.

The first step was to estimate the impact of relative deprivation on health. The results showed that the higher the relative deprivation, the worse the health status of the group, regardless of the comparison group or sex. This finding is consistent with those of most other studies (Eibner, Sturn & Gresenz, 2004; Eibner & Evans, 2005; Kondo et al., 2008, 2009; Subramanyam et al., 2009; Mangyo & Park, 2011). Because the first step in this analysis was consistent with the need for the mediating effect to be verified, we then estimated the influence of relative deprivation on the three unhealthy behavior variables (smoking, alcohol drinking and betel nut chewing). The results showed that the greater the relative deprivation caused by unequal income in men, the greater the degree of these three behaviors in each comparison group. For women, the relative deprivation was only correlated with the probability of current smoking for the educational level comparison group, whereas for alcohol drinking and betel nut chewing, the results were similar to those in men. Regardless of the comparison group, the greater the relative deprivation, the higher the probability of current alcohol drinking and betel nut chewing. Finally, the effects of relative deprivation, along with the three unhealthy behaviors, were simultaneously incorporated into an empirical model to verify whether the three unhealthy behaviors had complete or partial mediation. The results showed differences between men and women. When the male comparison group was set as educational level, area of residence and age + educational level, there was no significant correlation between relative deprivation and health, whereas smoking, alcohol drinking and betel nut chewing had significantly negative effects on health. This implies that these negative health behaviors had a complete mediating effect on relative deprivation and health status. When all subjects and age groups were compared, although relative deprivation had an effect on health, its influence decreased compared with the estimation derived in the first step. This result suggests that the three unhealthy behaviors had some mediating effect on relative deprivation and health status. For women, smoking had a partial mediating effect only when the comparison object was educational level. As for alcohol drinking and betel nut chewing, when individuals felt relative deprivation compared with those of the same age group, educational level and age + educational level, alcohol drinking and betel nut chewing had a complete mediating effect.

It is evident that smoking had a greater impact in men than in women in the role of mediating effect, implying that when men feel relative deprivation due to lower income than others, they are more likely to obtain relief from their dissatisfaction through smoking, thus affecting their health. For women, smoking behavior had a complete mediating effect only when their income was lower than that of those with the same educational level, which also suggests that smoking behavior acts as an important transmission channel between relative deprivation and health status for men compared with women. As for alcohol drinking and betel nut chewing, their mediating effect is similar in men and women. When all subjects were compared, alcohol drinking and betel nut chewing had partial mediation in the process of health impacts caused by income inequality. When the comparison groups were educational level, area of residence and age + educational level, relative deprivation caused by income inequality had complete mediating effect on both men and women. In short, smoking, alcohol drinking and betel nut chewing all had mediating effects on men, whether partial or complete. For women, only alcohol drinking and betel nut chewing had mediating effects, whereas the mediating effect of smoking in women was insignificant. Because of the traditional gender roles in Hainan’s culture, most men shoulder a relatively heavy household financial burden. Relative deprivation due to income inequality leads to the relieving of pressure and dissatisfaction not only through alcohol drinking and betel nut chewing but also through smoking. The low smoking rate among women may be related to the traditionally conservative culture, in which Hainan women may think that their smoking will be viewed differently than smoking by men. Therefore, smoking had almost no mediating effect on women, but alcohol drinking and betel nut chewing had significantly higher mediating effects.

The influence of these three behaviors among men was partial mediation when the comparison group was defined as all subjects and age. However, when the comparison groups were set as educational level, area of residence and age + educational level, all three unhealthy behaviors had a complete mediating effect. This suggests that if two men have the same educational level or both live in an urban area, the man with the lower income will feel deprived or experience greater work and life pressures, due to living in an urban area. Therefore, he is prone to obtaining relief from his dissatisfaction through tobacco, alcohol and betel nuts consumption. As for women, regardless of the setting of the comparison group, the mediating effect of alcohol and betel nuts was significantly greater than that of tobacco, indicating that, due to the relative deprivation caused by uneven income, their dependence on alcohol and betel nuts was greater than on tobacco.

We further performed an analysis directly using income for comparison rather than the Yitzhaki Index. Compared to those with high income, men with the lowest category of income (ranging from RMB 0 to 5,999 yuan) were more likely to smoke, drink alcohol and chew betel nuts, thus affecting their health. As for women, smoking had partial mediation only in those who earned no income, whereas the mediating effect of alcohol drinking and betel nut chewing was the same as that in men. The mediating effect was partial in those who with no income and in those who earned RMB 1–5,999 yuan. Comparing the results of using income for direct comparison or using the Yitzhaki Index, the mediating effect when comparing income directly was obviously smaller. Individual relative deprivation is a subjective feeling. In order to quantify this subjective feeling, the Yitzhaki Index provides a mathematical formula for relative deprivation constructed by taking individuals as reference points. Analysis based on this index can better reveal the negative psychology of being deprived among those who are relatively poor due to uneven income distribution. This may negatively affect health through health risk behaviors such as smoking, alcohol drinking and betel nut chewing.

However, this study has certain limitations. First, relative deprivation caused by income inequality was calculated based on the average monthly income of individuals in the past year, whereas individuals usually compare their own income with others without considering the structure or distribution of the family income (Subramanyam et al., 2009). However, in the 2017 HISHIR, data on personal income were collected as ranked data; thus, the real average income of individuals could not be known. Because of this limitation of the data, only the median value of income interval could be used as the actual income. Therefore, relative deprivation calculated by this method would not be consistent with the real income. Second, because the empirical data cannot provide information on personal subjective feelings, the relative deprivation feeling obtained by the Yitzhaki Index may differ from an individual’s true feelings. As in the existing literature, this study assumes that the degree of relative deprivation perceived as a subjective personal feeling is similar to that of the relative deprivation feeling calculated by the Yitzhaki Index. Third, this study sets up five types of comparison groups and assumes that individuals will compare themselves to other people in the same comparison group; however, individuals may not know the actual income of other people. Nevertheless, the Yitzhaki Index calculated in this study is derived from the assumption that individuals know the income of the comparison objects. Finally, this study assumed that the greater the relative deprivation, the more the consumption of tobacco, alcohol and betel nuts, thus affecting health. Because the database used in this study provided cross-sectional data, it was difficult to prove a causal relationship between relative deprivation and the behaviors of smoking, alcohol drinking and betel nut chewing. Future research should use time-series data to verify causality.

## Conclusions

This study examined whether relative deprivation caused by income inequality was transferred to the consumption of tobacco, alcohol and betel nuts through dissatisfaction, anger, pressure and other negative emotions, and further led to worse self-perceived health status. The harmful effects of tobacco, alcohol and betel nuts on health have long attracted the attention of the Chinese government. For example, the government has imposed a higher tax rate on tobacco, banned smoking in public places, penalized drunk driving, publicized the physiological toxicity of betel nuts and adopted other related policies to curb people’s consumption. However, if the phenomenon of income inequality cannot be improved, the relative deprivation caused by income inequality may cause people to turn to negative health behaviors to obtain relief from the relative deprivation. Therefore, the government should pay focused attention to the factors of income inequality when formulating relevant policies.

## Supplemental Information

10.7717/peerj.8728/supp-1 Supplemental Information 1 Surveyed data summary.

10.7717/peerj.8728/supp-2 Supplemental Information 2 Original data and basic information.

10.7717/peerj.8728/supp-3 Supplemental Information 3 Data codebook and questionnaire.

## Additional Information and Declarations

### Data Availability

The following information was supplied regarding data availability: The raw data is available in the Supplemental Files.
