# Supplementary material for: Relationship between relative deprivation and health of Hainan Island residents: mediating effect of negative health behaviors
Source: PeerJ. 2020 Mar 24;8:e8728. doi: 10.7717/peerj.8728 (PMC7100593; doi:10.7717/peerj.8728)
Supplement: Supplemental Information 3 [file peerj-08-8728-s003.pdf]

1. Gender:
   1= Males, 2= Females;
2. Age (years):
   1= 25–34 years, 2= 35–44 years, 3=45–54 years, 4= 55–64 years;
3. Marital status:
   1= With partner, 0=Without partner;
4. Educational level:
   1= Primary school, 2= Junior high school, 3= Normal high school or technical secondary school, 4= Junior college or undergraduate;
5. Chronic diseases:
   1= With chronic diseases, 0= Without chronic diseases;
6. Area of residence:
   1= Rural area, 2= Township, 3= County, 4= Urban area;
7. Average monthly income:
   0= no income, 1= less than RMB 1500 yuan, 2= RMB 1500 yuan to less than RMB 3000 yuan, 3=RMB 3000 yuan to less than RMB 4500 yuan, 4= RMB 4500 yuan to less than RMB 6000 yuan, 5= RMB 6000 yuan to less than RMB 7500 yuan, 6= RMB 7500 yuan to less than RMB 9000 yuan, 7= RMB 9000 yuan to less than RMB 15,000 yuan, 8= RMB 15,000 yuan to less than RMB 21,000 yuan, 9=RMB 21,000 yuan and above;
8. Self-perceived health status:
   1= Not good, 2= Normal, 3= Good, 4= Very good, 5= Excellent;
9. Smoking:
   0= Never smoke, 1= Once smoked but not smoking now, 2= Smoking now but not every day, 3= Smoking every day now;
10. Alcohol drinking:
    0= Never drink, 1= Once drank but have not drunk in the past year, 2= Have drunk in the past year but no addiction, 3= Have drunk in the past year and possibly have addiction;
11. Betel nuts chewing:
    0= Never chew, 1= Once chewed but not chewing now, 2= Chewing now but not every day, 3= Chewing every day now.
